# Optimizing Mass Rearing of the Egg Parasitoid, *Telenomus podisi*, for Control of the Brown Stink Bug, *Euschistus heros*

**DOI:** 10.3390/insects14050435

**Published:** 2023-05-03

**Authors:** Letícia Martins Parra, José Romário de Carvalho, William Wyatt Hoback, Regiane Cristina de Oliveira

**Affiliations:** 1 Crop Protection Department, School of Agronomic Sciences, São Paulo State University “Júlio de Mesquita Filho” (FCA/UNESP), Botucatu 18610034, SP, Brazil; parra.mleticia@gmail.com (L.M.P.); regiane.cristina-oliveira@unesp.br (R.C.d.O.); 2 Department of Natural Sciences, State Secretary of Education of Espírito Santo, Guaçui 29560000, ES, Brazil; jromario_carvalho@hotmail.com; 3 Noble Research Center, Department of Entomology and Plant Pathology, Oklahoma State University, Stillwater, OK 74078, USA

**Keywords:** biological control, stink bug, parasitoids, mass rearing, optimization

## Abstract

**Simple Summary:**

Biological control programs are used to effectively manage pests in a more sustainable way, such as the case of the microwasp parasitoid Telenomus podisi for managing eggs of the brown stink bug Euschistus heros, a key pest in South American soybean. A biological control program requires continuous production of the parasitoids and thus, artificial diets and freezing of host eggs are necessary. We evaluated the parasitism capacity of *T. podisi* reared on fresh or previously frozen eggs from *E. heros* fed on natural or two artificial diets. Seven constant temperatures were tested for each condition. We measured biological parameters of wasps and the rates of parasitism. We found that 24 °C had better parasitism and viability parameters, and was most-favorable to *T. podisi* production. We also found that both tested artificial diets for *E. heros* produced eggs that support mass rearing of this egg parasitoid.

**Abstract:**

The parasitoid *Telenomus podisi* Ashmead, 1893 is used in biological control programs in Brazil against eggs of *Euschistus heros* (Fabricius, 1798), a key pest of soybean, *Glycine max* (L.) Merr. To optimize the mass production of parasitoids, artificial diets and storage of host eggs at low temperatures have been developed; however, direct comparisons of the effects of these conditions have not occurred. We assessed a double factorial arrangement composed of six treatments (fresh or cryopreserved *E. heros* eggs from adults fed on natural or two artificial diets). We evaluated the biological characteristics and parasitism capacity of *T. podisi* produced from these treatments across seven temperatures. The thermal range between 21 and 30 °C resulted in satisfactory daily parasitism in all treatments tested, with an inverse relationship between temperature and female survival. The best parasitoid biological parameters were found between 21 and 27 °C, where all tested diets supported *T. podisi* development, with the best results from artificial diets. Fresh eggs and those frozen in liquid nitrogen and stored at −196 °C until use supported parasitoid development. These results suggest that the best method to mass rear *T. podisi* is to use artificial diets to rear *E. heros* and store eggs until needed, and then rear parasitoids at 24 °C.

## 1. Introduction

The brown stink bug, *Euschistus heros* (Hemiptera: Pentatomidae), is one of the key pests in soybean, *Glycine max* (L.) Merr., grown in Brazil. As a piercing-sucking insect, it is able to feed on both the plant and the grains, which can cause indirect damage to the plants and direct damage to yields [1]. It is a neotropical pentatomid and due to the increase in global temperature in the past decades, *E. heros* attacks soybean crops across Brazil [2].

The majority of producers still exclusively use chemical control for managing *E. heros* [3]. Although chemical control is one of the pillars of Integrated Pest Management (IPM), it should be used as the last management tactic, always based on pest monitoring, and following the recommended dose and rotating mode of action. Unfortunately, the traditional culture of the producers and the lack of different compounds for the management of pentatomids has resulted in overuse of chemical pesticides and widespread insecticide resistance [4]. In addition, over reliance on chemical pesticides has caused a decrease in natural enemies, environmental damage, and an increase in production costs [5,6].

The use of biological control has been increasing for pest management in crops as a way to reduce the use of pesticides and increase the sustainability of the agroecosystem, while preventing economic damage [7]. The egg parasitoid *Telenomus podisi* (Hymenoptera: Scelionidae) has been shown to be effective in managing populations of *E. heros*, the preferred host [8]. By parasitizing the egg stage, it has advantages in preventing the establishment and feeding damage of the pest [9]. Currently, around 60,000 hectares of soybean in Brazil are managed for *E. heros* with the release of *T. podisi*, with potential for expansion in the coming years [3,10].

For the release of *T. podisi* to occur effectively, a sufficient amount of parasitoids must be produced in specialized biofactories. However, the production of *T. podisi* requires substantial labor since the shelf life of natural enemies is low. An additional challenge is that, in most cases, the parasitoids are used at specific times of the year, depending on the planting cycle of each crop [11,12]. Large-scale insect rearing aims to reduce costs and increase production without reducing parasitoid quality [13,14]. Currently, techniques using artificial diets [15,16,17] and storage of host eggs at low temperatures [11,18,19] help in reducing rearing costs. Parasitoids also require hosts eggs with good quality and sufficient nutritional value [20]. Consequently, the egg aspect and content of nutrients in mass rearing are essential for parasitism, providing enough parasitoids for field release. 

The development of research in quality control of *T. podisi* is still new in Brazil, as the commercialization of this parasitoid was only registered in 2019 [21]. The reproductive rates of parasitoids are the basis for evaluating the control potential of a species as a biological control agent applied with inundative releases in the field [22]. Biological parameters, including sex ratio and viability, can also affect the success of parasitism in the field [23]. In addition, climate conditions strongly influence insect physiological development, migration, and dispersal [24]. Therefore, studies to optimize rearing of *T. podisi* based on biological attributes and rearing temperature are essential to understand the main requirements of this natural enemy, improving mass production with satisfactory quality to manage pests in the field [23]. 

The objectives of this study were to evaluate the parasitism capacity, thermal requirements, and biological characteristics of *T. podisi* that emerged from *E. heros* eggs. Eggs were obtained from adult stink bugs fed with different diets and used fresh or stored eggs that were frozen in liquid nitrogen. All conditions were tested at different temperatures (15 ± 2, 18 ± 2, 21 ± 2, 24 ± 2, 27 ± 2, 30 ± 2, and 33 ± 2 °C). Our work provides new information for *T. podisi* rearing to assist in the success of biological control programs against *E. heros* in Brazil and elsewhere. 

## 2. Materials and Methods

### 2.1. Rearing of E. heros with Natural and Artificial Diets

The rearing of *E. heros* was carried out at the Laboratory of the Research Group on Integrated Pest Management in Agriculture (AGRIMIP), located at the School of Agricultural Sciences (FCA/UNESP) in the municipality of Botucatu (22°50′42″ S 48°26′03.9″ W), maintained under controlled conditions of light (14 h of photophase), relative humidity (70 ± 10%), and temperature (25 ± 2 °C).

Adult and third-stage nymphs of *E. heros* were kept in plastic cages (25 × 25 × 12 cm) covered with voile fabric to allow for ventilation. Raw cotton strips were provided in the adult cages as substrate for copulation, walking, and oviposition. As a food source, a natural or artificial diet was provided to the insects, depending on the bioassays to be performed. The natural diet consisted of 10 green bean pods (*Phaseulus vulgaris* L.) and 20 grains of raw peanuts (*Arachis hypogaea* L.) per cage. In artificial diet cages (Table 1), 10 pieces of 1 cm^2^ of the diet were offered in each box. The artificial diets were created by grinding the ingredients in a grain grinder and, in sequence, in a homemade processor. The ingredients were homogenized and the diets were placed into aluminum foil, and then baked in an oven at 60 °C for 3 h to dry. As a source of water, cotton soaked in filtered water was offered in the rearing cages.

Every 2 days, the eggs of *E. heros* were removed from the cages and placed in plastic dishes (60 × 12 mm) for hatching of the nymphs, to maintain the laboratory colony or were used for the bioassays. For comparisons with fresh eggs, 1 mL of eggs (1000 eggs on average) from each type of diet was wrapped in aluminum foil, closed with adhesive tape, and cryopreserved in liquid nitrogen (−196 °C) until the beginning of the bioassay.

### 2.2. Rearing of T. podisi

The rearing of *T. podisi* was carried out in the same place as the rearing of *E. heros*, under the same controlled laboratory conditions. About 5 mL of fresh *E. heros* eggs were glued with nontoxic white glue onto cardboard (8 × 10 cm), placed in 3-liter plastic pots, covered with plastic film and elastic, allowing for parasitism for 5 days. As a food source, pure honey was rubbed on the sides of the pots. This procedure was carried out once a week to continue the rearing of *T. podisi* and to supply parasitoid females for bioassays.

### 2.3. Parasitism Capacity and Thermal Requirements of T. podisi in E. heros Eggs from Different Diets

To assess the parasitism capacity of *T. podisi*, a paper card (1 × 7 cm) was created with 20 eggs of *E. heros* for each replication of the six treatments described below. Cards were transferred to glass tubes (2 × 8 cm). Each treatment had five replications, forming a double factorial scheme, with eggs from six different conditions (natural and two artificial diets, each cryopreserved or fresh) tested at seven different temperatures: 15 ± 2, 18 ± 2, 21 ± 2, 24 ± 2, 27 ± 2, 30 ± 2, and 33 ± 2 °C. The treatments consisted of: T1: Fresh eggs of *E. heros* from natural diet; T2: Cryopreserved eggs of *E. heros* from natural diet; T3: Fresh eggs of *E. heros* from artificial diet 1 (AD1); T4: Cryopreserved eggs of *E. heros* from AD1; T5: Fresh eggs of *E. heros* from artificial diet 2 (AD2); T6: Cryopreserved eggs of *E. heros* from AD2.

One female newly emerged and mated of *T. podisi* was added to each glass tube, a honey droplet was provided as a food source, and the tube was sealed with PVC plastic film. The card with the eggs from each replication of the treatments was changed each 24 h until the female died. When removed, the cards were placed in plastic bags (24 × 4 cm) at the respective temperature in Biochemical Oxygen Demand (BOD) climatic chambers. Parasitism was evaluated (Equation (1)) over the days by the total number of eggs and number of parasitized eggs, which were determined using a stereoscopic microscope (Leica EZ4). The development of *T. podisi* (egg-adult) was observed from the day of parasitism until emergence.

(1) $$\%\;\mathrm{Parasitism}=\left(\mathrm{no}.\;\mathrm{parasitized}\;\mathrm{eggs}\times 100\right)÷\mathrm{total}\;\mathrm{no}.\;\mathrm{of}\;\mathrm{eggs}$$

**Figure 1:**
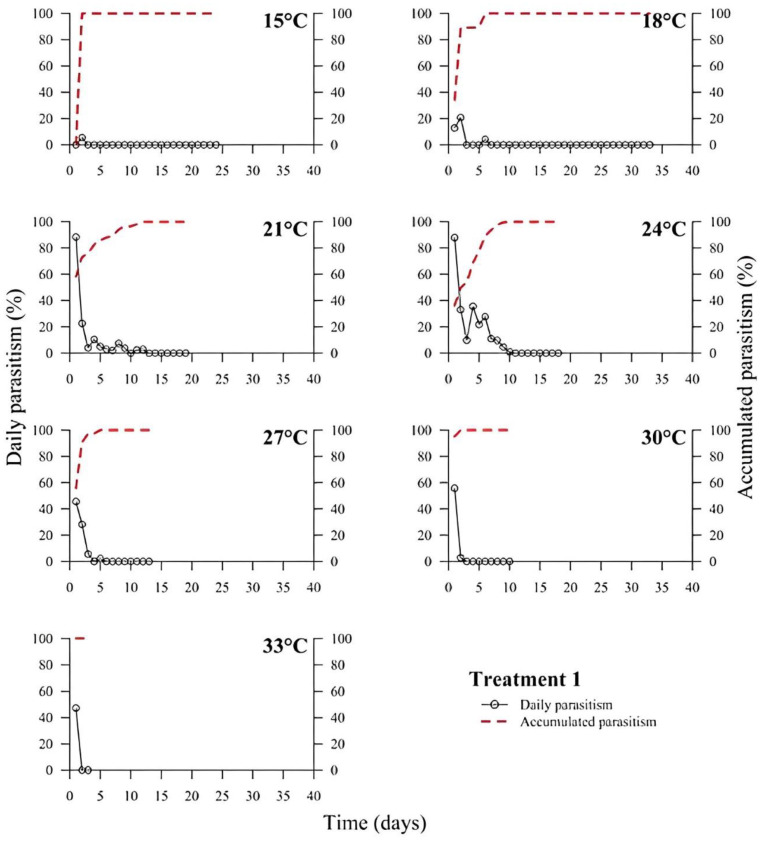
Daily and accumulated parasitism (%) of *Telenomus podisi* of *Euschistus heros* fresh eggs reared with natural diet (Treatment 1) at different temperatures under 70 ± 10% relative humidity and 14:10 h of photoperiod.

**Figure 2:**
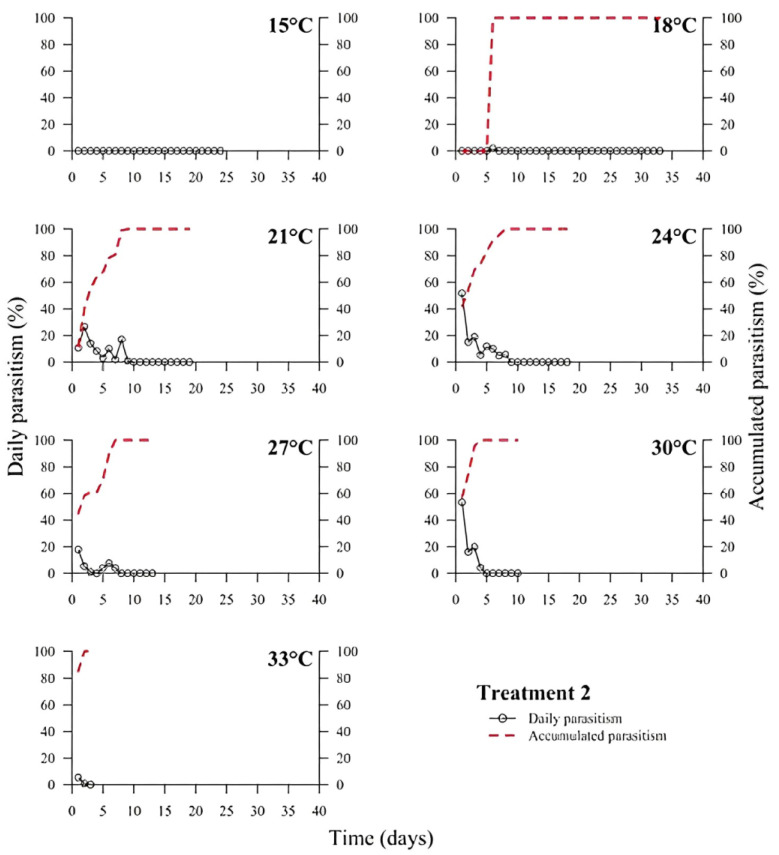
Daily and accumulated parasitism (%) of *Telenomus podisi* of *Euschistus heros* cryopreserved eggs reared with natural diet (Treatment 2) at different temperatures under 70 ± 10% relative humidity and 14:10 h of photoperiod.

**Figure 3:**
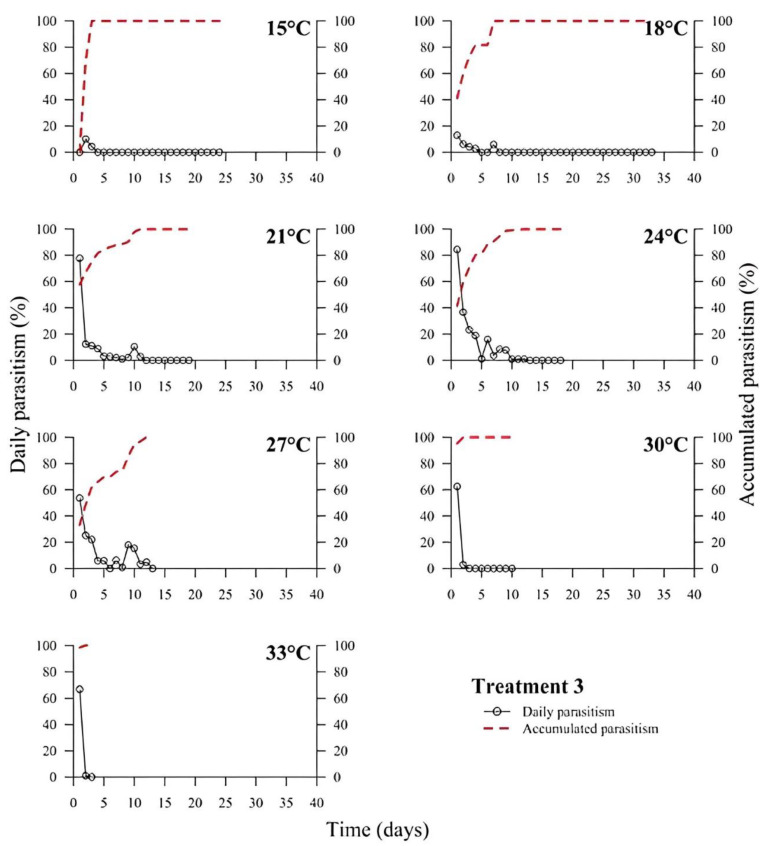
Daily and accumulated parasitism (%) of *Telenomus podisi* of *Euschistus heros* fresh eggs reared with artificial diet 1 (Treatment 3) at different temperatures under 70 ± 10% relative humidity and 14:10 h of photoperiod.

**Figure 4:**
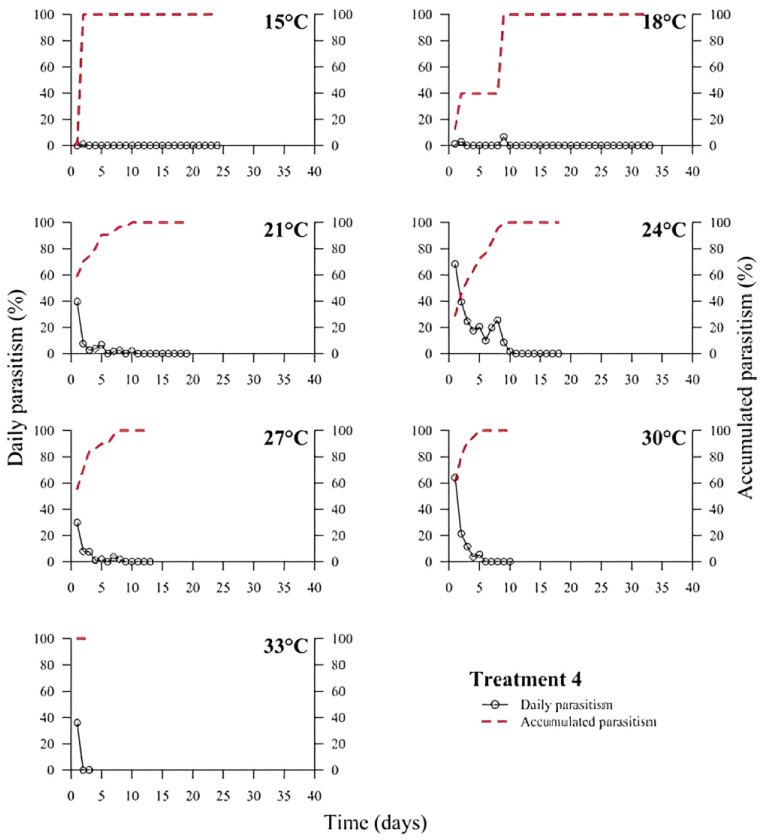
Daily and accumulated parasitism (%) of *Telenomus podisi* of *Euschistus heros* cryopreserved eggs reared with artificial diet 1 (Treatment 4) at different temperatures under 70 ± 10% relative humidity and 14:10 h of photoperiod.

**Figure 5:**
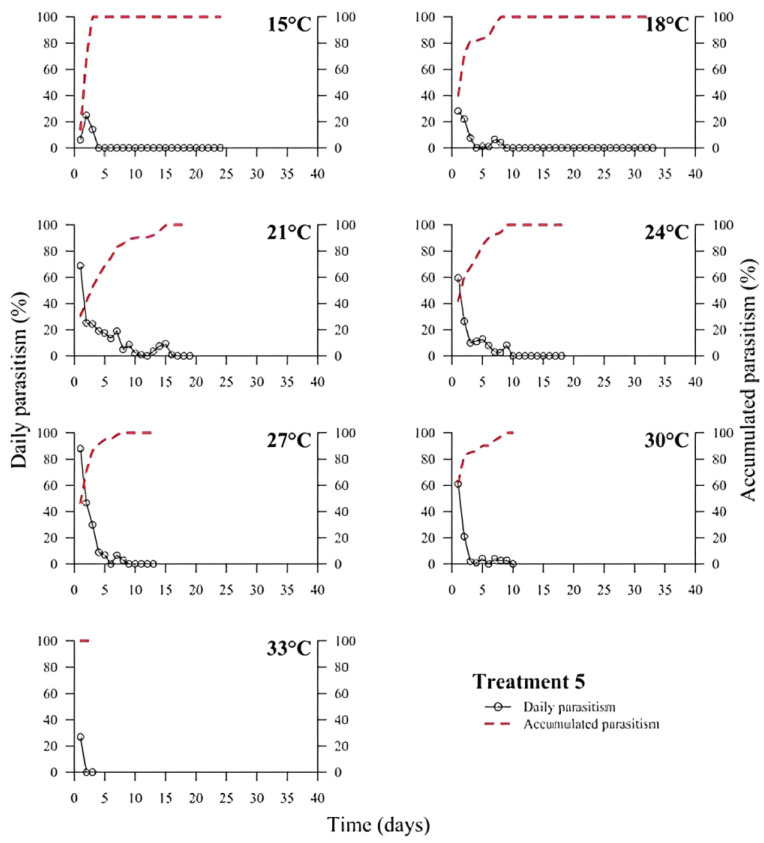
Daily and accumulated parasitism (%) of *Telenomus podisi* of *Euschistus heros* fresh eggs reared with artificial diet 2 (Treatment 5) at different temperatures under 70 ± 10% relative humidity and 14:10 h of photoperiod.

**Figure 6:**
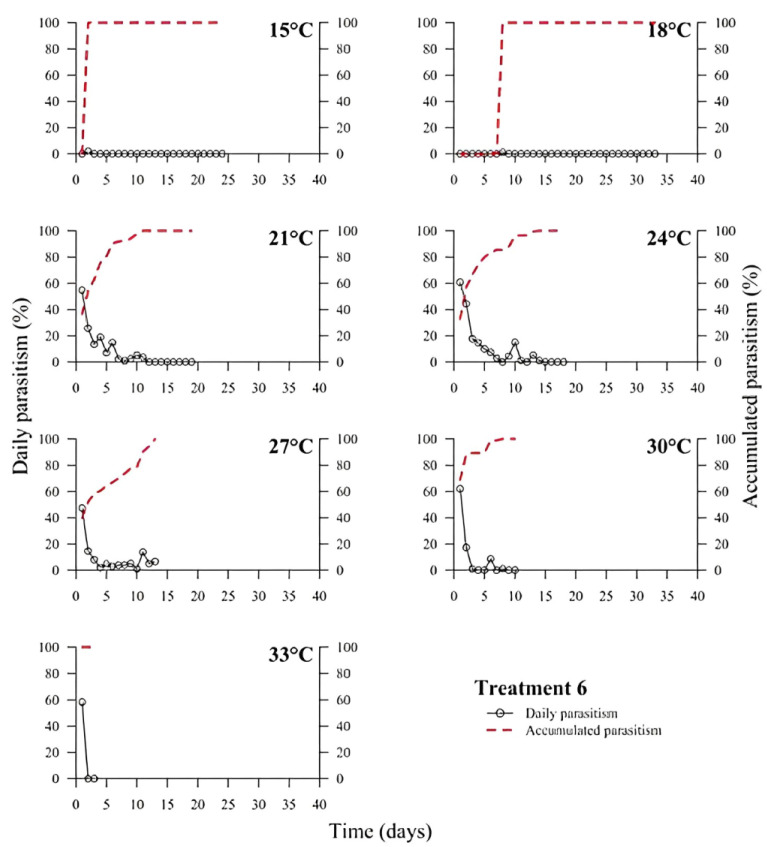
Daily and accumulated parasitism (%) of *Telenomus podisi* of *Euschistus heros* cryopreserved eggs reared with artificial diet 2 (Treatment 6) at different temperatures under 70 ± 10% relative humidity and 14:10 h of photoperiod.

The thermal requirements for *T. podisi* development in each treatment were based on the parameters of thermal constant (*K*) and the lower temperature threshold (*To*), given by the development period time (egg-adult). *T. podisi* development rates as a function of temperature were calculated using both a linear and a nonlinear model (Briere 1) (Table 2) and the parameters were estimated with the Levenberg–Marquardt method using the minpack.lm package in R 3.5 [25,26]. The most suitable models were selected based on the chi-square test of adherence (χ^2^), the logarithm of maximum likelihood (LogLik), the sum of squared residuals (RSS), Akaike’s information criterion (AIC), the Bayesian information criterion (BIC), and the adjusted coefficient of determination (adjR^2^). The daily parasitism and accumulated parasitism (calculated by adding daily parasitism rates until the female’s death) were based on the percentage of parasitized eggs (Equation (1)) per day in each tested temperature until female *T. podisi* death (Figure 1, Figure 2, Figure 3, Figure 4, Figure 5 and Figure 6).

### 2.4. Biological Parameters of T. podisi in E. heros Eggs from Different Diets

The effect of the different diets offered to *E. heros* and storage conditions of eggs on the biological parameters of *T. podisi* were evaluated by placing one female parasitoid newly emerged and mated in a glass tube (2 × 8 cm) containing a paper card (1 × 7 cm) with 30 *E. heros* eggs and six replications. The treatments were composed of the same diets and egg conditions as the parasitism capacity bioassay (T1 to T6), and were also evaluated at seven different temperatures (15 ± 2 to 33 ± 2 °C).

A honey droplet was offered in each glass tube as a food source. After 24 h of parasitism at 25 ± 2 °C, the cards with parasitized eggs of *E. heros* were removed and placed in plastic bags (24 × 4 cm) at the respective temperatures in BOD climatic chambers. After emergence, 15 adult females and 15 adult males of *T. podisi* from the eggs in each treatment were stored in 70% alcohol until body length was measured using the software Leica Application Suite-Version 1.6.0. The total number of eggs, number of parasitized eggs, number of eggs with emergence holes, and number of females and males emerged were quantified. With these data, it was possible to evaluate the following biological parameters: Parasitism (Equation (2)), viability (Equation (3)), number of individuals per egg (Equation (4)), and sex ratio (Equation (5)).

(2) $$\mathrm{Parasitism}=\left(\mathrm{no}.\;\mathrm{parasitized}\;\mathrm{eggs}\times 100\right)÷\mathrm{total}\;\mathrm{no}.\;\mathrm{of}\;\mathrm{eggs}$$

(3) Viability=no. eggs with emergence hole÷no. parasitized eggs

(4) No. individuals per egg=total number of individuals ÷no. of eggs with emergence hole

(5) Sex ratio=n females÷no. females+males

We carried out a completely randomized exploratory analysis using a 6 × 7 factorial arrangement (diets × temperatures). The means of body length of females and males of *T. podisi* and the means of parasitism, viability, number of individuals per egg, and sex ratio were tested for homogeneity [29], normality, and independence of variances [30].

To determine the influence of treatments and temperatures, we used a generalized linear model with a Gaussian distribution for the body length parameter, binomial residual distribution for the sex ratio and viability parameters, and a Poisson residual distribution for the parameters of the number of individuals per egg and parasitism, followed by Tukey’s multiple comparison test (*p* < 0.05). All statistical analyses were performed using R 3.5 [26].

## 3. Results

### 3.1. Parasitism Capacity of T. podisi in E. heros Eggs from Different Diets

At low temperatures (15 and 18 °C), there was a low or no daily parasitism in the first days of all experiments (T1 to T6). The longest survival of *T. podisi* females in this thermal range was 25 to 35 days, with accumulated parasitism reaching a plateau over time (Figure 1, Figure 2, Figure 3, Figure 4, Figure 5 and Figure 6). At 33 °C (highest temperature), a low daily parasitism was also observed in the first days, except for T3 that reached 60% of eggs parasitized. Females also had the lowest survival times of 4 days. Moreover, at 33 °C, no accumulated parasitism was observed. 

The thermal range of 21 to 24 °C was the most suitable for *T. podisi*, with T1 and T3 reaching more than 80% parasitism with other treatments exhibiting 30 to 70% parasitism. The maximum parasitism observed at 27 and 30 °C was 60%, with the exception of T5 at 27 °C, which had more than 80% parasitism. In all treatments, we observed that increases in temperature decreased the rate of daily and accumulated parasitism over time and reduced female survival (Figure 1, Figure 2, Figure 3, Figure 4, Figure 5 and Figure 6).

### 3.2. Thermal Requirements of T. podisi in E. heros Eggs from Different Diets

Based on the models’ fit tests, the R^2^ adjustment was the most suitable for the tested parameters. As shown in Table 2 and Table 3, the *To* values of each treatment are better fit by the nonlinear model where they are higher than the *To* obtained by the linear model.

The thermal constant (*K*) had high variation among treatments, with lower values being observed in T1, T4, and T6 and the highest value in T2 (Table 4). In contrast, adjusted R^2^ values were higher for T1, T6, T3, and lower for T2 (Table 4). Treatments composed of artificial diets and fresh eggs (T3 and T5) had higher *K* values compared with those composed of artificial diets and cryopreserved eggs (T4 and T6).

**Figure 7:**
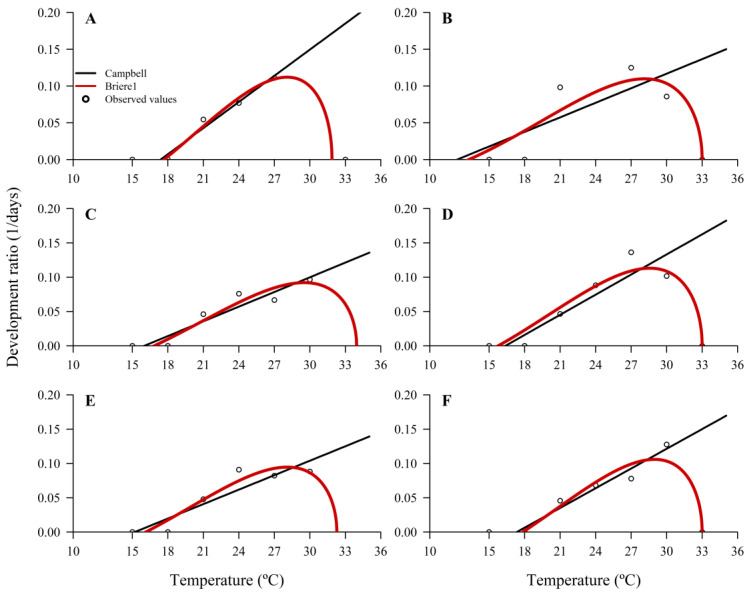
Linear and nonlinear regression models of temperature-dependent ratio of development for *Telenomus podisi*. (**A**–**F**) correspond to the treatments T1 to T6 respectively.

In general, the upper maximum temperature (*T_max_*) for survival and parasitism ranged from 32 to 34 °C in all treatments (Table 3 and Figure 7). The optimum temperature (*T_opt_*) had low variation among egg treatments, with values ranging from 28 to 29 °C (Table 3 and Figure 7). However, the lowest minimum temperature (*T_min_*) had different values among treatments, with higher values being observed in treatments with fresh eggs (T1 and T3) when compared to cryopreserved eggs (T2 and T4), with the exception of treatments T5 and T6 (Table 3 and Figure 7). All treatments composed of cryopreserved eggs (T2, T4, and T6) resulted in the *T_max_* of 33 °C (Figure 7)

### 3.3. Biological Characteristics of T. podisi in E. heros Eggs from Different Diets

There was a significant factorial interaction between eggs from different diets and temperature on body length in females (F = 2.970; df = 15,232; *p* = 0.000) and males (F = 1.350; df = 10,100; *p* = 0.040) of *T. podisi* (Table 5 and Table 6, respectively). For the females, T2 and T5 did not affect size in relation to the tested temperatures. Treatments subjected to temperatures of 27, 30, and 33 °C also did not show significant differences in body length (Table 5). Body length of *T. podisi* males only showed differences among treatments based on tested temperatures, with differences between T5 and T6 (Table 6). The thermal range between 24 and 27 °C produced the largest body sizes in both females and males.

At 15 and 18 °C, no insects developed. At 33 °C, the parasitoids only emerged from T5 and T6 diets and were only females (Table 5 and Table 6).

There was a significant factorial interaction between diets and temperature for *T. podisi* parasitism (F = 4.121; df = 30,168; *p* < 0.001). Overall, the thermal range from 21 to 30 °C showed the best results (Table 7), where 24 °C had better results numerically. At temperatures of 15, 18, and 21 °C, T5 (AD2 and fresh eggs) had statistically better results than the other treatments (Table 7). At other temperatures, treatments composed of artificial diets (T3, T4, T5, and T6) also showed better parasitism results, except at 30 °C. At this temperature, T4, T5, and T6 (artificial diets) and T2 (natural diet with cryopreserved eggs) had similar rates of parasitism (Table 7).

In contrast, viability (F = 55.5807; df = 6205; *p* < 0.0001 and F = 4.2023; df = 5205; *p* = 0.0012 for temperature and diet, respectively), number of individuals per egg (F = 18.5349; df = 6205; *p* < 0.0001 and F = 1.7163; df = 5205; *p* = 0.1323), and sex ratio (F = 19.2542; df = 6205; *p* < 0.0001 and F = 2.9260; df = 5205; *p* = 0.0142) did not show a significant factorial interaction. Similar to parasitism, extreme low (15 and 18 °C) and high temperatures (33 °C) resulted in lower biological performance of the parasitoids, with better results observed in the thermal range from 21 to 30 °C (Table 8, Table 9 and Table 10). The temperature of 24 °C provided the best viability parameter, as well as parasitism rate. In terms of viability and sex ratio, the artificial diets provided better performance compared to the natural diet (Table 8 and Table 10). In the number of individuals per egg parameter, the diets did not influence the results (Table 9).

## 4. Discussion

The interaction between parasitoid and host depends on many factors, with temperature and egg quality being among the most important parameters that influence parasitism and survival [23,24,31,32]. Temperature directly influences life process in small insects [33]. Our parasitism capacity results show an inverse relationship between temperature and survival period. As observed in most insects, the increase in temperature results in a decreased lifespan. This can be explained by increases in the insect’s metabolic rate at high temperatures and decreased metabolism at low temperatures [34,35]. Similar effects of high temperatures on survivorship and parasitism capacity were found for several other parasitoids species [34,36,37,38,39,40]. 

In the cases of applied inundative biological control programs, the parasitism capacity is more relevant than the period of time females remain alive in the field [39]. In all treatments, the highest rate of parasitism occurred in the first days (Figure 1, Figure 2, Figure 3, Figure 4, Figure 5 and Figure 6). This can be explained by the ability of *T. podisi* to store mature eggs in the ovaries or oviducts, completing oogenesis even before the adult emerges from the host egg [41].

According to the International Organization for Biological Control (IOBC) guidelines, ≥80% is accepted as a good parameter of parasitism for the genus *Trichogramma* [42]. Based on that, the goal of 80% of parasitism was not achieved in most treatments, except for T5 at 27 °C. Species, such as *T. podisi,* are unable to perform lipogenesis and depend entirely on lipid storage in the larval stage for fecundity and lifespan in the adult stage [33,39,43,44]. In addition, since they cannot synthesize lipids in the adult stage, parasitoids are more vulnerable to temperature increases and pesticides than most pests, including the host *E. heros* [39,43,45]. Therefore, nutritional aspects are essential for the success in parasitism, and parasitoids tend to choose sources that provide greater energy return [20,46]. 

Our results showed that artificial diets had better parasitism rates at 15, 18, and 27 °C than treatments with natural diets. These data emphasize the importance of studies on the influence of eggs from hosts reared with artificial diets in the production of parasitoids. Possibly, eggs from artificial diets had superior nutritional quality, resulting in greater parasitism under stressful conditions (thermal extremes), which has not been reported in other studies with artificial diets that examined the biology of *T. podisi* [16,47]. Future host preference tests and field bioassays should be carried out, in addition to the analysis of egg nutrition, influenced by diet and storage.

Rearing for biological control release also depends on providing parasitoids when host eggs are deposited. The efficiency and development of *T. podisi* are temperature-dependent, allowing for some storage of adults, and the ability to increase or decrease development [24,32]. From thermal requirement data, given by the thermal constant *K* (degree-days), upper and lower temperature threshold (*T_max_* and *T_min_*), and optimal temperature (*T_opt_*), it is possible to estimate the influence of temperature on the parasitoid’s lifespan [48,49], making it possible to optimize the mass production of natural enemies. The hosts (*E. heros*) and the parasitoids used in this bioassay originate from colonies kept in the laboratory for years, in which the hosts are reared under the same conditions as T1 (natural diet and fresh eggs). Possibly, since they are adapted to this type of diet, they needed less thermal accumulation to complete development and future studies should examine the effects of temperature on successive generations.

The host–parasitoid interaction can be influenced by associative learning over generations, where parasitoids adapt and prefer certain types of hosts due to pre-emergence conditioning established during their larval period [50,51,52]. *E. heros*, the preferred host for *T. podisi*, has a *K* of 327 degree-days [35], which is significantly higher than all treatments. This is a good indication that, in favorable situations in the field, the parasitoid will complete its cycle faster than the host, further contributing to pest management [34,53].

Values of *K*, *T_min_*, *T_max_*, and *T_opt_* vary according to the type of host used, species of parasitoid, and adjusted model for the analyses, potentially relating our results to those of other studies [22,34,35,54,55,56,57]. Since T2 presented *T_min_* of 16 °C and was composed of a natural diet (little variety of ingredients) and cryopreserved eggs, the nutritional aspect of the eggs from this diet was potentially inferior compared to the other treatments, influencing *T_min_*.

Quality control parameters are essential for mass rearing of insects. This is especially challenging for biological control insects that must be produced as needed to match the crop and target pest. The ability to maintain laboratory colonies and quickly produce the desired number of individuals requires the creation of artificial diet, ways to store host eggs for long periods, and the ability to accelerate or slow down development. These manipulations must not impact the overall quality of the reared insects [7,23,39]. In Europe, there are quality control guidelines for more than 30 species of natural enemies according to the IOBC, but the majority are species not produced in Brazil [42]. However, these guidelines serve as a model for national quality control tests [58,59].

Based on the analysis of biotic factors, including adult size and life history, across rearing temperatures, we can assess the quality of *T. podisi* for release in the field [60], since the success of this parasitoid release depends on these quality parameters [23,39]. Even though we found significant factorial interaction between the diets and the temperatures tested, there are no expressly significant differences between body length based on the diet and cryopreservation of eggs offered to *T. podisi*. Body size may be relevant in reducing energy costs [61,62]. Larger insects may reduce movements to search for hosts, are likely to forage in a more heterogeneous environment, interacting with more species, and exhibit greater preferential prey selection than smaller individuals [61,62,63,64]. As only females are responsible for parasitism in the field and differences were found only in body length of females at 21 °C, these data can be useful to improve the success of the parasitoid in biological control programs at this temperature. 

Temperature was important in the rearing of *T. podisi*, since survival was prolonged at lower temperatures. Low temperatures can reduce the insect’s metabolic rate, affecting the viability of parasitized eggs, consequently preventing insect emergence [34,57,65]. The loss of turgidity of the eggs as the temperature increases can prevent the insertion of the parasitoid ovipositor into the egg’s chorion, reducing successful parasitism [39,66].

Considering the release of parasitoids in the field, these results may indicate an important factor in applied biological control programs due to increases in average global temperature, mainly in regions with high thermal amplitude (low temperatures at night and high during the day). Thermal extremes can be harmful to the survival, development, dispersion, and longevity of parasitoids, compromising the efficiency of parasitoid release and management of target pests [24]. With the increase in global temperature, our results of the upper temperature threshold are relevant for mass releases of *T. podisi* in regions with high temperatures, considering that *T_opt_* ranged between 28 and 29 °C. Parasitism rates of 60% were found when *T. podisi* was reared at 27 and 30 °C, demonstrating that *T. podisi* is capable of parasitizing more than half of pentatomid eggs soon after release at higher field temperatures [39,43]. Increases in average global temperature tend to negatively affect the distribution of parasitoids more than of hosts, due to lower *T_opt_* and *T_max_* [54,67,68]. In an ideal scenario, delaying the development of parasitoids when field temperatures border on the upper threshold can help in the successful application of biological control. Our results can be useful for biological programs in areas with thermal ranges from 21 to 30 °C, indicating that *T. podisi* is adapted to these conditions, although field experiments must confirm our results. 

The nutritional composition of the egg is also fundamental for the choice and development of the parasitoid [69]. Since AD2 is rich in different types of grains (sunflower seed and wheat germ) compared to the AD1 and the natural diet (Table 1), the *E. heros* eggs obtained from AD2 likely had a higher nutritional value than the other diets, allowing for the development of parasitoids at high temperatures (33 °C). Future nutritional analysis of diets and eggs should be performed to confirm this assumption. In addition, the production cost of each diet should be analyzed to conclude whether the shift from natural to artificial diet is beneficial.

The artificial diets, especially AD2 with fresh eggs (T5), showed better results than the natural diet, contradicting other studies in which artificial diets did not influence the evaluated parameters of *T. podisi* [16,47]. Viability and sex ratio had better results with fresh eggs than cryopreserved eggs, possibly due to small changes in the physical-nutritional characteristics caused by low temperatures, causing females to prefer fresh eggs [20]. In order to increase fitness, foraging theory predicts that parasitoids, when searching for host eggs, evaluate and explore the energy content before parasitizing the egg [20,46,70,71]. Several studies have been carried out on the use of cryopreservation in mass rearing of egg parasitoids [11,72,73,74,75]. Although the use of liquid nitrogen to store host eggs is beneficial to maintain a stock throughout the year, our results demonstrated that cold storage affected the biological performance of *T. podisi*.

## 5. Conclusions

Based on our results, *T. podisi* can be mass reared for release in tropical areas. We conclude that the thermal range between 21 and 30 °C presented satisfactory daily and accumulated parasitism in all treatments tested. Similarly, the biological parameters evaluated (body length, parasitism, viability, number of individuals/egg, and sex ratio) showed that rearing temperatures between 21 and 30 °C are favorable for the development of *T. podisi*. Within this thermal range, all tested diets were favorable for the rearing of the parasitoid from the host with AD2 providing the best quality of parasitoids. As parasitism and viability presented better results at 24 °C and egg parasitoids are generally reared at 25 ± 2 °C in Brazil, we recommend 24 °C to mass rear *T. podisi* using fresh or cryopreserved *E. heros* eggs from adults provided AD2.

## Figures and Tables

**Table 1:** Type of diet, ingredients, and quantity offered per box in the rearing of *Euschistus heros*.

Diet	Ingredients	Quantity Offered Per Box
Natural	Fresh bean pod (*Phaseolus vulgaris* L.)	10 units
	Raw peanut (*Arachis hypogaea* L.)	20 g
Artificial 1	35 g raw peanut	10 units of 1 cm^2^
	30 g soybean (*Glycine max* L.)	
	5 g sucrose	
	25 mL distilled water	
	2 mL anticontaminants ^1^	
Artificial 2	35 g raw peanut	10 units of 1 cm^2^
	30 g fresh bean pod	
	10 g sunflower seed (*Helianthus annuus* L.)	
	10 g wheat germ (*Triticum* spp.)	
	10 g soybean	
	5 g sucrose	
	2 mL anticontaminants ^1^	

^1^ Anticontaminants composed of tetracycline (0.00765 ppm), nipagim (10,000 ppm), and sorbic acid (800 ppm) [17].

**Table 2:** Linear and nonlinear models used to estimate the ratio of development to room temperature (24 ± 2 °C).

Model	Equation	Definition of Variables	References
Linear	$r\left(T\right)=a+b\cdot T$	*a*: line intercept *b*: line slope	[27]
Briere 1	$r\left(T\right)=a\cdot T\cdot \left(T-T_{min}\right)\cdot {\left(T_{max}-T\right)}^{\frac{1}{2}}$	*T_min_*: minimum temperature *T_max_*: maximum temperature	[28]

**Table 3:** Nonlinear regression model parameters of temperature-dependent development ratio, lower temperature threshold (*T_min_* °C), upper temperature threshold (*T_max_* °C), and optimal temperature threshold (*T_opt_* °C) for *Telenomus podisi* under laboratory conditions (14 h of photophase, 70 ± 10% RH, and 25 ± 2 °C).

Treatment	Model	Parameters	Estimates	SE
T1	Briere1	*a*	1.974 × 10^−4^	9.490 × 10^−5^
		*T_max_*	31.80	4.573
		*T_min_*	17.70	9.624 × 10^−1^
		*T_opt_*	28.10	
T2	Briere1	*a*	1.192 × 10^−4^	6.734 × 10^−5^
		*T_max_*	33.00	3.696 × 10^−4^
		*T_min_*	13.28	6.090
		*T_opt_*	28.10	
T3	Briere1	*a*	1.170 × 10^−4^	7.176 × 10^−5^
		*T_max_*	33.95	4.680
		*T_min_*	16.85	2.381
		*T_opt_*	29.50	
T4	Briere1	*a*	1.469 × 10^−4^	2.495 × 10^−5^
		*T_max_*	33.00	1.635 × 10^−4^
		*T_min_*	15.76	1.332
		*T_opt_*	28.60	
T5	Briere1	*a*	1.377 × 10^−4^	3.937 × 10^−5^
		*T_max_*	32.28	1.593
		*T_min_*	16.13	1.259
		*T_opt_*	28.1	
T6	Briere1	*a*	1.637 × 10^−4^	3.287 × 10^−5^
		*T_max_*	33.00	1.796 × 10^−4^
		*T_min_*	17.84	1.353
		*T_opt_*	29.00	

**Table 4:** Linear regression model parameters and R^2^ adjusted for temperature-dependent development ratio, lower temperature threshold (*To* °C), and thermal constant (*K* °day) for *Telenomus podisi* under laboratory conditions (14 h of photophase, 70 ± 10% RH, and 25 ± 2 °C).

Treatments	*a*	SE	*b*	SE	*To*	*K*	R^2^aj
T1	−0.206138	0.033938	0.011857	0.001492	4.85	84.34	0.9539
T2	−0.081186	0.126882	0.006602	0.005186	13.32	151.46	0.4475
T3	−0.114140	0.042444	0.007135	0.001742	8.76	140.15	0.7978
T4	−0.160032	0.074011	0.009775	0.003037	6.25	102.30	0.7006
T5	−0.106663	0.058089	0.007022	0.002383	9.38	142.41	0.6575
T6	−0.166039	0.030716	0.009581	0.001260	6.02	104.37	0.9342

**Table 5:** Body length (mm) of *Telenomus podisi* females emerged from eggs of *Euschistus heros* reared from different diets at seven temperatures. Means followed by the same lower case letters in columns and upper case letters in rows do not differ at the 5% level.

Females Body Length						
Temperature (°C)	ND ^1^		AD1 ^2^		AD2 ^3^	
	Fresh ^4^ (T1)	Cryo ^4^ (T2)	Fresh (T3)	Cryo (T4)	Fresh (T5)	Cryo (T6)
15	-	-	-	-	-	-
18	-	-	-	-	-	-
21	0.998 ± 0.029 bC	-	1.064 ± 0.014 aAB	1.119 ± 0.016 aA	1.032 ± 0.013 aBC	1.089 ± 0.017 aA
24	0.981 ± 0.011 bB	1.022 ± 0.011 aAB	1.042 ± 0.007 aA	1.026 ± 0.015 bAB	1.024 ± 0.012 aAB	1.012 ± 0.025 bA
27	1.110 ± 0.050 aA	1.027 ± 0.017 aA	1.049 ± 0.007 aA	1.031 ± 0.014 bA	1.048 ± 0.008 aA	1.060 ± 0.018 abA
30	1.020 ± 0.010 abA	0.947 ± 0.000 aA	1.053 ± 0.017 aA	1.033 ± 0.016 bA	1.064 ± 0.012 aA	1.037 ± 0.015 abA
33	-	-	-	-	1.079 ± 0.019 aA	1.083 ± 0.025 abA

^1^ Natural diet. ^2^ Artificial diet 1. ^3^ Artificial diet 2. ^4^ Fresh or cryopreserved eggs of *Euschistus heros* parasitized by *Telenomus podisi*. “-” represents insufficiency of numerical data to perform statistical analysis.

**Table 6:** Body length (mm) of male *Telenomus podisi* emerged from eggs of *Euschistus heros* reared from different diets at seven temperatures. Means followed by the same lower case letters in columns and upper case letters in rows do not differ at the 5% level.

Males Body Length						
Temperature (°C)	ND ^1^		AD1 ^2^		AD2 ^3^	
	Fresh ^4^ (T1)	Cryop ^4^ (T2)	Fresh (T3)	Cryop (T4)	Fresh (T5)	Cryop (T6)
15	-	-	-	-	-	-
18	-	-	-	-	-	-
21	-	-	0.992 ± 0.014 aA	1.012 ± 0.029 aA	1.000 ± 0.022 bA	1.042 ± 0.017 abA
24	0.972 ± 0.021 A	0.991 ± 0.021 aA	0.982 ± 0.014 aA	0.980 ± 0.015 aA	1.028 ± 0.024 abA	0.974 ± 0.013 bA
27	-	1.085 ± 0.055 aA	1.020 ± 0.000 aA	-	1.020 ± 0.000 abA	1.040 ± 0.009 abA
30	-	0.996 ± 0.035 aA	1.060 ± 0.015 aA	1.070 ± 0.000 aA	1.060 ± 0.014 aA	1.059 ± 0.014 aA
33	-	-	-			

^1^ Natural diet. ^2^ Artificial diet 1. ^3^ Artificial diet 2. ^4^ Fresh or cryopreserved eggs of *Euschistus heros* parasitized by *Telenomus podisi*. “-” represents insufficiency of numerical data to perform statistical analysis.

**Table 7:** Parasitism (%) of *Telenomus podisi* emerged from eggs of *Euschistus heros* reared from different diets under seven temperatures. Means followed by the same lower case letters in columns and upper case in rows do not differ at the 5% level.

Parasitism						
Temperature (°C)	ND ^1^		AD1 ^2^		AD2 ^3^	
	Fresh ^4^ (T1)	Cryop ^4^ (T2)	Fresh (T3)	Cryop (T4)	Fresh (T5)	Cryop (T6)
15	1.20 ± 0.49 eB	0.00 dC	2.79 ± 0.75 dB	0.19 ± 0.20 eB	8.99 ± 1.34 efA	0.40 ± 0.28 eB
18	7.59 ± 1.23 dB	0.40 ± 0.28 cC	6.39 ± 1.13 dB	2.40 ± 0.69 eC	14.20 ± 1.69 deA	0.20 ± 0.20 eC
21	30.39 ± 2.47 bB	18.20 ± 1.91 aC	26.99 ± 2.32 bB	13.59 ± 1.65 bcC	44.79 ± 2.99 aA	29.80 ± 2.44 abB
24	48.40 ± 3.11 aA	24.80 ± 2.23 aC	40.60 ± 2.85 aA	47.00 ± 3.07 aA	28.39 ± 2.38 bcBC	37.00 ± 2.72 aAB
27	16.40 ± 1.81 cCD	8.00 ± 1.26 bE	32.39 ± 2.55 abAB	10.8 ± 1.47 cdDE	37.99 ± 2.76 abA	23.80 ± 2.18 bcBC
30	11.59 ± 1.52 cdC	18.80 ± 1.94 aAB	13.19 ± 1.62 cBC	21.19 ± 2.06 bA	19.79 ± 1.99 cdAB	18.00 ± 1.90 cdABC
33	9.59 ± 1.39 cdABC	1.20 ± 0.49 cD	13.59 ± 1.65 cA	7.20 ± 1.20 dBC	5.39 ± 1.04 fC	11.4 ± 1.51 dAB

^1^ Natural diet. ^2^ Artificial diet 1. ^3^ Artificial diet 2. ^4^ Fresh or cryopreserved eggs of *Euschistus heros* parasitized by *Telenomus podisi*.

**Table 8:** Viability (%) of *Telenomus podisi* emerged from eggs of *Euschistus heros* reared from different diets. Means followed by the same upper case letters in “Diet” and lower case in “Temperature” do not differ at the 5% level.

	Viability		
Diet		Temperature (°C)	
T1	12.33 ± 0.038 B	15	0 d
T2	14.1 ± 0.038 B	18	0 d
T3	22.4 ± 0.046 AB	21	27.6 ± 4.6 b
T4	20.5 ± 0.049 AB	24	66.3 ± 4.9 a
T5	28.4 ± 0.05 A	27	19.9 ± 3.6 b
T6	20.3 ± 0.046 C	30	16.7 ± 2.6 bc
		33	3.8 ± 2.4 cd

**Table 9:** Number of individuals per egg of *Telenomus podisi* emerged from eggs of *Euschistus heros* reared from different diets under seven temperatures. Means followed by the same upper case letters in “Diet” and lower case in “Temperature” do not differ at the 5% level.

	Number of Individuals per Egg		
Diet		Temperature (°C)	
T1	0.339 ± 0.080 A	15	0.000 c
T2	0.360 ± 0.083 A	18	0.000 c
T3	0.606 ± 0.131 A	21	0.777 ± 0.08 a
T4	0.454 ± 0.083 A	24	0.883 ± 0.06 a
T5	0.617 ± 0.113 A	27	0.687 ± 0.08 a
T6	0.588 ± 0.151 A	30	0.93 ± 0.21 a
		33	0.083 ± 0.05 b

**Table 10:** Sex ratio (female to male) of *Telenomus podisi* emerged from eggs of *Euschistus heros* reared from different diets under seven temperatures. Means followed by the same upper case letters in “Diet” and lower case in “Temperature” do not differ at the 5% level.

	Sex Ratio		
Diet		Temperature (°C)	
T1	0.299 ± 0.07 AB	15	0.000 b
T2	0.200 ± 0.061 B	18	0.000 b
T3	0.414 ± 0.071 AB	21	0.530 ± 0.078 a
T4	0.327 ± 0.069 AB	24	0.657 ± 0.066 a
T5	0.442 ± 0.088 A	27	0.538 ± 0.082 a
T6	0.255 ± 0.07 B	30	0.409 ± 0.099 a
		33	0.083 ± 0.047 b

## Data Availability

All datasets used or analyzed during this study are included in this article.
